# Supplementary material for: Cortical dynamics of neural-connectivity fields
Source: J Comput Neurosci. 2025 Apr 10;53(2):373–91. doi: 10.1007/s10827-025-00903-8 (PMC12181116; doi:10.1007/s10827-025-00903-8)
Supplement: Supplementary file 1 — (zip 138 KB) [file 10827_2025_903_MOESM1_ESM.zip › Notations_and_Index_for_NC_JCN_review.pdf]

1 Parameters for LFP Simulation in Multi-Layer Cortex

Multi-Layer cortical connectivity is defined in equations 1.

$$\begin{aligned} \alpha &= \frac{g\partial_t A_t^2 T^2}{2m} = 0.5 \\ m &= 10 \\ C &= 1 \end{aligned} \tag{1}$$

The resulting distribution of the LFP in layer 1 and 2 is given in equation 2, Fig. 4.

$$\begin{aligned} \phi_1(t) &= H(t)\frac{1}{m}\sin(10t)\sin(\frac{t}{2})\exp^{-\frac{t}{2}} \\ \phi_2(t) &= H(t)\frac{1}{m}\sin(10t)\cos(\frac{t}{2})\exp^{-\frac{t}{2}} \end{aligned} \tag{2}$$

2 Notations and Mathematical Terms

Nomenclature

Analysis and Calculus

α, β, γ Scalar constant; real or complex valued

$\boldsymbol{r}, \boldsymbol{r_0}$ Point on the cortical surface

$\boldsymbol{S}, \boldsymbol{W}$ Vector or matrix valued function

$\boldsymbol{S}^{-1}$ Inverse of the function $\boldsymbol{S}$

$\delta(x)$ Dirac delta function

$\int, \iint$ Integral

$\mathcal{L}$ Lagrangian function, real-valued

μ, ν, η Greek indices for time and space coordinates

∇ Gradient operator

∇^2 Laplacian operator

∂_μ Partial derivative of the μth coordinate

∂_i Partial derivative of the ith space coordinate

ϕ^* Complex conjugate of ϕ

c Speed of wave propagation

dA Integral measure

$H(t)$ Heaviside function

i The imaginary unit ($i^2 = -1$)

i, j, k Latin indices for space coordinates

m Mass term in Klein-Gordon field

t_{ret} Retarded time, taken at the time of propagation of the signal

a, b, c Scalar constant; real or complex valued

Differential Geometry

$\boldsymbol{\partial} - i\epsilon\boldsymbol{A}$ Covariant derivative

$\boldsymbol{\phi}$ Vector/Tensor variable; real or complex valued

$\boldsymbol{D}$ Covariant derivative

$\boldsymbol{F}, f$ Smooth functions defined over the cortical surface (e.g. manifold)

ϵ, g Coupling constants between neural and connectivity fields

$\partial_t^2 - \sum_i \partial_i^2$ d'Alembert operator

∂^2 d'Alembert operator

ϕ, ψ Scalar variable; real or complex valued

$A_\mu A^\nu$ Repeated indices are summed, Einstein notation

$F_{\mu\nu}$ Tensor of rank 2

$g^{\mu\nu}$ μth and νth component of the metric

$G_{\mu\nu}$ Einstein tensor

R Ricci scalar

$R_{\mu\nu}$ Ricci tensor
